# Disparity Analysis for Cardiac Surgical Outcomes: The Citizenship Factor

**DOI:** 10.3390/jcdd10070292

**Published:** 2023-07-09

**Authors:** Mohammad F. Babgi, Haitham M. Albar, Mohammed H. Miny, Haitham Alzahrani, Mohammad Shakil Ahmad, Riyaz Ahmed Shaik, Elnazeer O. Ahmed

**Affiliations:** 1 Department of Cardiac Surgery, King Abdullah Medical City, Makkah 57657, Saudi Arabia; 2 Department of Surgery, College of Medicine, Majmaah University, AlMajmaah 11952, Saudi Arabia; 3 Department of Family & Community Medicine, College of Medicine, Majmaah University, AlMajmaah 11952, Saudi Arabia

**Keywords:** health care disparities, coronary artery bypass, Saudi Arabia

## Abstract

(1) Background: Disparity in clinical care on the basis of gender, socioeconomic status, ethnic and racial variation is an established phenomenon. The focus on health disparities was led on by the report of the Secretary’s Task Force on Black & Minority Health, which emphasized that the burden of death and illness was in excess among black people and other minorities. In Saudi Arabia, cardiac health care is being provided to a heterogeneous group of patients during pilgrimage time. This mixed population comprises different socio-economic backgrounds, demographics, ethnicities and languages. This study was carried out to assess for any disparities in cardiac surgical outcomes after isolated CABG surgery between Saudi citizens and non-Saudi patients. (2) Methods: The data of 2178 patients who underwent isolated coronary artery bypass surgery at King Abdullah Medical City from December 2014 to July 2020 were extracted. Patient demographics, clinical features, comorbidities, diagnoses, surgical procedures, complications, length of hospital stay and mortality were included in the data. The primary outcome was mortality after coronary artery bypass grafting surgery. (3) Results: A total of 2178 isolated CABG procedures were conducted during the study period with almost 57.5% of patients being Saudi citizens in comparison with 42.5% of non-Saudi citizens. The male gender represented the majority of the population, with a total of 1584 patients, representing 72.7% of the total study population. The rate of mortality had no statistical significance with the mortality rate of 5% vs. 5.3% (*p* < 0.786). The postoperative morbidities were comparable for all the parameters except for postoperative extracorporeal membrane oxygenation (ECMO). (4) Conclusions: In the present study, the chances of survival and postoperative outcomes are not associated with nationality per se, but with underlying comorbidities.

## 1. Introduction

Universal health coverage is one of the targets of sustainable development goals [1]. The pillars of universal health coverage are accessibility, affordability, availability and quality of health care services. Of the various factors studied and researched, disparity in health care is one of the major hindrances in health and health care across different racial, ethnic and socio-economic groups within the population. However, it has also been advocated that disparity is not restricted to aforementioned factors, but also includes lifestyle choices, age, sexual orientation, lack of access and personal, socio-economic and environmental characteristics [2].

The focus on health disparities was led on by the report of the Secretary’s Task Force on Black & Minority Health issued by the former US Health and Human Services Secretary, Margaret M. Heckler [3]. It was emphasized in the report that the burden of death and illness was in excess among black people and other minority Americans than the nation’s population as a whole. Six conditions were taken into account in the report, which were cancer, heart disease and stroke, diabetes, infant mortality, cirrhosis and homicide and accidents. It reported 86% of excess mortality in black people. It was emphasized that approximately 45% of deaths up to the age of 70 years in the black population could have been averted if better evaluation, detection and treatment had been available [4]. 

Similarly, a clear discrepancy in the cardiac catheterization rates, angioplasty and coronary artery bypass grafting was observed in black minority patients compared to their white counterparts in the Veteran Health system in the US. Black people were less likely to undergo invasive cardiac procedures [5]. Besides racial disparity in health care utilization, urban–rural disparity owing to household income was found to be a significant factor in preventive services utilization, as demonstrated in a study conducted in China using the China Health and Nutrition Survey in 2011 [6]. It is evident that the disparity in health care services is more pronounced due to a clear disparity in health insurance utilization among minority communities. As a consequence, they tend to utilize fewer preventive services, suffer poorer health outcomes, higher mortality and disability rates. Therefore, from the evidence garnered, it becomes very obvious that health care disparities are universal and inevitable. 

The inequalities in health care services for invasive cardiac procedures among different nationalities is minimally studied and presents a gap in the literature [7,8,9,10]. Several factors contribute to the higher prevalence of CVD in the Saudi population. One significant factor is lifestyle choices, including a high-fat diet, a lack of physical activity and smoking. Age distribution also plays a role in the disparities in the cardiac health of Saudi and non-Saudi people. These factors are more prevalent in the Saudi population, leading to a higher incidence of CVD.

The current Saudi population is 36.6 million, comprising 57.6% males and 42.3% females, with a majority (51.45%) belonging the age group of 25–54, with 11.3% being above 55 years of age [11]. The world population differs in these age distributions when compared to Saudi people, e.g., in Europe, the population of people above 65 years of age is higher (17%), and in Africa, the geriatric population is only 3% [12]. The GDP of Saudi countries is USD 29,922 in 2023 [13].

Talking about obesity and cardiovascular risk factors, a study in Saudi Arabia shows that the prevalence of obesity (BMI ≥ 30) is 24.7%, which is quite high than the world population (13%) [14], and it is significantly associated with type 2 diabetes (odd ratio, (OR) = 1.52), hypercholesterolemia (OR = 1.69), and hypertension (OR = 1.61) [15]. Another national survey conducted between 1995 and 2000 observed an overall prevalence of obesity among Saudi adults to be 35.6%. The smoking prevalence is roughly 14% among the Saudi population, which is less than the prevalence of smoking in the world population (32%) [16,17]. The meat consumption in Saudi Arabia is 54 kg per person per year, which is less than the US average (124 Kg per person per year), but quite more than most other countries [18].

Moreover, there are genetic and physiological differences between the Saudi and non-Saudi population that may contribute to the disparity in cardiac health.

The prevalence of coronary artery disease in Saudi Arabia is reportedly 5.5%, and it accounts for 45% of total deaths reported [19]. Saudi Arabia holds a religious importance, and the Holy City of Makkah is visited for ritual practices by citizens with varied backgrounds during the pilgrimage seasons. Also, apart from pilgrimage, the non-Saudi work force accounts for about 30% of the total population [20]. This mixed population comprises different socio-economic backgrounds, demographics, ethnicities and languages.

Taking all these factors into account, it can be assumed that there is a clear difference in the modifiable and non-modifiable risk factors of cardiovascular diseases among the Saudi and non-Saudi populations, which might alter the results of a cardiac surgical procedure.

Hence, we intend to study the potential disparity in the surgical management of patients with coronary artery disease. 

The outcome of isolated coronary artery bypass grafting (CABG) procedures is well known; thus, isolated (CABG) cases are studied. We aim to assess for any disparities in cardiac surgical outcomes after isolated CABG surgery between Saudi citizens and non-Saudi citizens. 

## 2. Materials and Methods

For the purpose of this analysis, data were extracted from the cardiac surgery department database at King Abdullah Medical City for the period of December 2014 to July 2020 for all patients who underwent isolated coronary artery bypass surgery. A total of 2178 patients underwent an isolated CABG procedure during the specified period and were included using total enumeration. Patients who underwent other cardiac procedures either isolated from or combined with CABG were excluded from the study.

Patient demographics, clinical features, comorbidities, diagnoses, surgical procedures, complications, length of hospital stay and mortality were all included in the data. This information was converted into electronic data-collecting forms using a coding scheme, with no nominative information included.

The primary study outcome was mortality after the index isolated coronary artery bypass grafting (CABG) surgery, and the secondary outcome was morbidity in terms of prolonged ventilation, reintubation, acute kidney injury, renal replacement therapy, re-exploration, new postoperative atrial fibrillation (AF), cerebrovascular accident (CVA), perioperative myocardial infarction (MI), sepsis, mediastinitis, postoperative intra-aortic balloon pump (IABP) and postoperative extracorporeal membrane oxygenation (ECMO). 

### Statistical Analysis

A cohort of 2178 patients who underwent isolated coronary artery bypass grafting (CABG) procedure were included in the study and retrospectively looked at the association of the nationality factor with mortality. The data were analyzed in Microsoft Excel and SPSS version 25. Continuous variables were presented in terms of means and standard deviations or medians and interquartile ranges depending upon skewness and kurtosis. Unadjusted association between categorical variables were assessed using chi-squared test. Unadjusted associations of continuous variables were assessed using the two samples, t-test or Wilcoxon rank test. Adjusted hazard ratios were computed using Cox proportional hazards regression analysis (stepwise forward LR), and the relationship between the covariates and mortality was assessed for the full cohort. Patients were assigned propensity scores and matched between Saudi and non-Saudi patients with key baseline characteristics such as gender, weight, height, Euro score, smoking history, dyslipidemia, obesity, diabetes mellitus, hypertension, cerebrovascular accidents and peripheral vascular diseases. Matched cohort was again checked for differences, which were identified as significant factors in Cox regression analysis. We analyzed key covariates that were selected a priori to study the effect of modifiers on the relationship between Saudi and non-Saudi citizens with all-cause mortality in the propensity-matched cohort.

Separate Kaplan–Meier survival analyses were carried out to compare the effect of nationality factor on mortality in the full and matched cohort. Binary logistic regression was then applied to assess the relationship between nationality and mortality outcome by adjusting the propensity-matched scores as covariates. All tests were performed using SPSS version 28. The study protocol was evaluated and approved by the institutional review board at King Abdullah Medical City with IRB number 22-943. 

## 3. Results

During the aforementioned period, isolated CABG procedures were conducted on 2178 patients. As evident from Table 1, a total of 1252 patients (57.5%) were Saudi citizens, in comparison to 926 patients (42.5%) who were non-Saudi citizens. The male gender represented the majority of the population with a total of 1584 patients, representing (72.7%) of the total study population. The two cohorts were comparable for the mean age, weight, ejection fraction and median European System for Cardiac Operative Risk Evaluation II (Euroscore II), which predicts the in-hospital mortality after cardiac surgery [21]. A history of diabetes mellitus, cerebrovascular accidents, peripheral vascular diseases and smoking was more commonly reported among Saudi patients than non-Saudi patients. Approximately, more than half of the patients belonged to class 2 of the New York Heart Association (NYHA) Classification. The CABG procedure was carried out as an elective procedure on almost 90% of patients, while around one-fifth of the total patients underwent emergency procedures.

As evident from Table 2, there was no statistical significance with regard to in-hospital mortality, with a mortality rate of 5% vs. 5.3% (P 0.786) among Saudi citizens and non-Saudi citizens, respectively. The postoperative morbidities of Saudi and non-Saudi patients were comparable for all the parameters except for postoperative ECMO.

Table 3 depicts the differences among the patients on the basis of mortality. A significant difference was observed on the basis of age and Euroscore II among patients who survived and those who did not with a *p* value of < 0.001. By comparing the comorbidities based on mortality status, diabetes mellitus was found to be significantly associated with the non-survivors (*p <* 0.011). Of the total mortality reported, 60% of patients were planned for an elective procedure, while approximately 37% had an emergency procedure. A significant difference between the mean values of creatinine clearance and ejection fraction was reported, with higher values associated with survivorship (*p* < 0.001). The distribution of the history of smoking in the patients who survived and those who did not was comparable (*p <* 0.122). 

A multi-variable Cox regression proportional hazard model was formulated to assess the risk of death associated with CABG procedure (Table 4). The predictors which were found to be significantly associated with death are Euroscore II, prolonged ventilation, reintubation, acute kidney injury, renal replacement therapy, mediastinitis, postoperative intra-aortic balloon pump (IABP), postoperative ECMO and total postoperative stay. A risk of fatal outcome that was four times greater was associated with prolonged ventilation and reintubation. Acute kidney injury and continuous renal replacement therapy were associated with a 3.39 and 2.19 hazard ratio, respectively. Those patients who had a postoperative intra-aortic balloon pump (IABP) and postoperative ECMO inserted had a 94% and 46% higher chance of mortality, respectively. 

The distribution of these significant covariates was observed among the matched cohort of Saudi and non-Saudi patients, and all the covariates were found to be comparable (Table 4).

**Figure 1:**
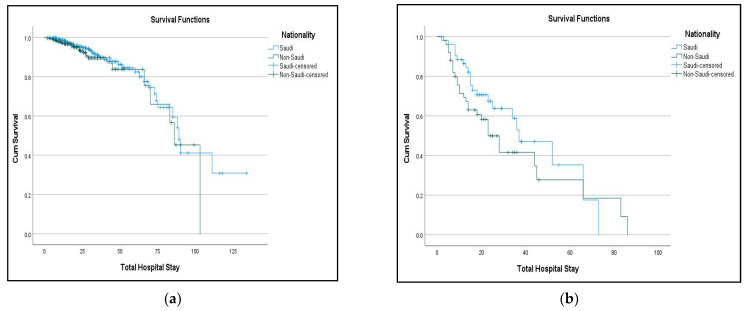
Comparison of survival among Saudi and non-Saudi patients (a = full cohort, b = matched cohort). (**a**) Log rank (Mantel–Cox): *p*-value = 0.109; (**b**) Log rank (Mantel–Cox): *p*-value = 0.202.

The survival curve depicts the probabilities of difference in the cumulative survival among Saudi and non-Saudi patients, and it is evident from the curve (Figure 1a) that the chances of survival among Saudi patients (median survival time: 89 months) are better than the non-Saudi patients (median survival time: 86 months). The survival curve was also formulated for the matched cohort (Figure 1b), and again, the Saudi patients (median survival time: 37 months) had better cumulative survival probabilities than the non-Saudi patients (median survival time: 23) (Figure 1).

Logistic regression was conducted to assess the odds of death based on nationality. The non-Saudi patients had odds of death that were 2.2 times higher (significant statistically) following the CABG procedure than the Saudi patients. The adjusted propensity score for all the variables favors Saudis (odds of 1.9), but has a wide confidence interval (0.306–12.085) and is also not statistically significant. (Table 5)

## 4. Discussion

As evident from our analysis, the mortality, morbidity and chances of survival are not associated with citizenship status per se. Rather, they were associated with the underlying comorbidities. While our study focuses on the Saudi and non-Saudi patients, various studies compared white people and non-white people in terms of CABG mortality and morbidity. 

Rumsfeld et al. (data from 1995 to 2001) found that black patients were more likely to have had a previous percutaneous coronary intervention, preoperative ECG ST-segment depression, higher blood creatinine, peripheral vascular disease, more likely to be current smokers, and receive preoperative diuretics at baseline. White patients, on the other hand, were more likely to have had previous cardiac surgery, lung disease and surgery. At 30 days, they found no difference in the unadjusted mortality between the black and white patients. They found that black patients were more likely than white patients to have immediate postoperative complications such as renal failure requiring hemodialysis, reoperation for bleeding and prolonged mechanical breathing [22]. Similarly, Maynard and Ritchie looked into veterans as well (29,918 subjects, eight percent black). They discovered that black CABG patients were younger, less likely to be married and less likely to receive internal mammary artery (IMA) grafts, and had higher levels of HTN (84 percent vs. 54 percent), diabetes (36 percent vs. 23 percent) and were active smokers (21 percent vs. 14 percent, all statistically significant). Black patients had a 9% greater risk of death (OR, 1.09; *p* = 0.01) and a 3% higher risk of rehospitalization (OR, 1.03; *p* = 0.02) two years after CABG. Gray et al. looked at long-term results in a single institution’s sample of 3728 isolated CABG patients. They discovered that at one year following surgery, black patients had a lower chance of survival than white patients, and this discrepancy expanded at five years. In addition to increased mortality, racial differences in the need for post-CABG readmission were identified [23].

In our present study, a significant difference was observed on the basis of age and Euroscore II among patients who survived and those who did not. This is also supported by the study by Hartz et al., which looked at 30-day operative mortality after isolated CABG (non-white patients, 11.9 percent). Patients who were non-white were younger and had a greater rate of renal failure and diabetes mellitus (*p <* 0.001). Meanwhile, non-white patients had a greater surgical mortality (up to 10% of projected operative mortality, *p <* 0.05), but there were no differences in the higher-risk subgroups [24]. The significant difference in the rates of survival could be attributed to the difference in language, which could act as a barrier in communication [10].

A nationwide survey conducted in 2001 in the US reports that health professionals are unaware of the existence of disparity in the health care system. Almost 65% of doctors had a perception that there is no difference in treatment between African Americans and white people [25]. Thus, the disparity can only be addressed if it is known to exist.

In our study, the predictors which were found to be significantly associated with death are Euroscore II, prolonged ventilation, reintubation, acute kidney injury, continuous renal replacement therapy, postoperative intra-aortic balloon pump (IABP), postoperative ECMO and total postoperative stay. A significantly higher proportion of non-Saudi patients required postoperative ECMO, which emerged as a predictor for survival in the current study. A convincing body of evidence also suggests that a prolonged duration of ECMO following cardiovascular surgery is associated with higher chances of mortality [26]. Although insignificant, the higher proportion of non-Saudi patients were found to develop postoperative complications. The Centre for Health Equities Research and Promotion model ascertains that the disparity in health and the health care system is influenced by an array of critical factors, which include individual, social, policy and provider-related factors [27].

Hannan et al. also looked for risk factors for early readmission after CABG surgery in the New York State database [28]. Within 30 days, 12.9 percent of eligible patients were readmitted for CABG-related reasons. Infection (28 percent) and heart failure (14 percent) were the most common reasons for readmission (16 percent) [19], while in a study by Bridges et al., many preoperative variables were found as significant predictors of surgical mortality using stepwise logistic regression. Patients with HTN, DM and heart failure were more likely to be younger, female and black [29]. 

Woods et al. found that diabetic patients experienced significantly more mortality than the patients without diabetes (RR, 1.67; *p* < 0.004), and diabetes treated with insulin (RR = 2.08) or oral agents (RR = 1.68), age (RR = 1.54), current smoker (RR = 1.72), former smoker (RR = 1.50), peripheral vascular disease (RR = 1.50) and HTN (RR = 1.40) were all independent predictors of cardiac mortality at 5 years (adjusted RR = 1.49, *p* = 0.001) [30,31]. 

The strength of the study lies in the fact that the cohort of Saudi and non-Saudi patients are comparable on background characteristics. Additionally, the propensity score is calculated to identify the independent predictor of death following an isolated CABG procedure. The limitations of our study are that it is retrospective in nature and was only focused on a single cardiac center, and in future planning projects, we should add cardiac centers of various regions in Saudi Arabia. 

## 5. Conclusions

The disparity effect for the outcomes post-CABG is well documented in the literature due to several reasons, especially because of health care access and eligibilities. In the present study, the chances of survival and postoperative outcomes are not associated with nationality per se, but with underlying comorbidities. 

The predictors of mortality associated with CABG are Euroscore II, prolonged ventilation, reintubation, acute kidney injury, continuous renal replacement therapy, postoperative intra-aortic balloon pump (IABP), postoperative ECMO and total postoperative stay. This study further paves the way for future research to further examine this ethical practical and universal issue of care inequality and identify the possible factors responsible for its occurrence.

## Figures and Tables

**Table 1:** Baseline characteristics and morbidities as per nationality (full cohort).

Characteristics		Saudi (n = 1252)	Non-Saudi (n = 926)	Total (N = 2178)	*p*-Value
**Socio-demographic characteristics**					
Gender	Male	869 (69.4%)	715 (77.2%)	1584 (72.7%)	<0.001
	Female	383 (30.6%)	211 (22.8%)	594 (27.3%)	
Age		55.4 ± 12.9	55.8 ± 11.6	55.5 ± 12.4	0.430
Weight		74.4 ± 15.7	74.3 ± 15.0	74.3 ± 15.4	0.940
Euroscore II		2.6 (2.1–4.1)	2.6 (2.1–4.3)	2.6 (2.1–4.1)	0.891
Ejection fraction (EF)		45.2 ±10.4	44.8 ± 10.3	45.05 ± 10.4	0.952
Smoker		493 (39.4%)	298 (32.2%)	791 (36.3%)	0.001
**New York Heart Association (NYHA) Classification**					
I		9 (0.7%)	5 (0.5%)	14 (0.6%)	0.090
II		786 (62.8%)	630 (68.0%)	1416 (65.0%)	
III		383 (30.6%)	234 (25.3%)	617 (28.3%)	
IV		65 (5.2%)	46 (5.0%)	111 (5.1%)	
**Comorbidities**					
Dyslipidemia		138 (11.0%)	86 (9.3%)	224 (10.3%)	0.188
Obesity		281 (22.4%)	205 (22.1%)	486 (22.3%)	0.865
Diabetes mellitus		820 (65.5%)	528 (57.0%)	1348 (61.9%)	0.000
Hypertension		849 (67.8%)	609 (65.8%)	1458 (66.9%)	0.316
Cerebrovascular accident		78 (6.2%)	34 (3.7%)	112 (5.1%)	0.008
Peripheral vascular disease		29 (2.3%)	10 (1.1%)	39 (1.8%)	0.031
Carotid disease		19 (1.5%)	14 (1.5%)	33 (1.5%)	0.991
Chronic obstructive pulmonary disease		58 (4.6%)	32 (3.5%)	90 (4.1%)	0.173
**Operative techniques**					
CABG on pump	Yes	1305 (95.9%)	56 (4.1%)	1361 (100%)	0.029
	No	761 (93.1%)	56 (6.9%)	817 (100%)	
	Total	2066 (94.9%)	112 (5.1%)	2178 (100%)	
CABG off pump	Yes	22 (100%)	0 (0.0%)	22 (100%)	0.251
	No	2044 (94.8%)	112 (5.2%)	2156 (100%)	
	Total	2066 (94.9%)	112 (5.1%)	2178 (100%)	
**Nature of the operation**					
Elective		1122 (89.6%)	771 (83.3%)	1893 (87%)	<0.001
Urgent		5 (0.4%)	5 (0.5%)	10 (0.5%)	
Emergency		123 (9.8%)	150 (16.2%)	273 (12.5%)	

**Table 2:** Mortality and morbidity incidence as per nationality (full cohort).

Postoperative Mortality and Morbidities	Saudi	Non-Saudi	Total	*p*-Value
In-hospital mortality	63 (5.0%)	49 (5.3)	112 (5.1%)	0.786
**Morbidities**				
Prolonged ventilation	101 (8.1%)	77 (8.3%)	178 (8.2%)	0.834
Reintubation	47 (3.8%)	31 (3.3%)	78 (3.6%)	0.614
New postoperative atrial fibrillation (AF)	236 (18.8%)	166 (17.9%)	402 (18.5%)	0.583
Re-exploration	66 (5.3%)	52 (5.6%)	118 (5.4%)	0.721
Acute kidney injury	64 (5.1%)	54 (5.8%)	118 (5.4%)	0.463
Continuous renal replacement therapy	64 (5.1%)	45 (4.9%)	109 (5.0%)	0.790
Postoperative cerebrovascular accident (CVA)	30 (2.4%)	16 (1.7%)	46 (2.1%)	0.284
Perioperative myocardial infarction (MI)	2 (0.2%)	2 (0.2%)	4 (0.2%)	0.762
Sepsis	73 (5.8%)	49 (5.3%)	122 (5.6%)	0.589
Mediastinitis	5 (0.4%)	4 (0.4%)	9 (0.4%)	0.907
Postoperative intra-aortic balloon pump (IABP)	79 (6.3%)	75 (8.1%)	154 (7.1%)	0.107
Postoperative ECMO	12 (1.0%)	19 (2.1%)	31 (1.4%)	**0.033**

**Table 3:** Baseline characteristics and comorbidities as per mortality outcome (full cohort).

Characteristics		Alive (n = 2066)	Dead (n = 112)	Total (N = 2178)	*p*-Value
**Socio-demographic characteristics**					
Gender	Male	1514 (95.6%)	70 (4.4%)	1584 (100%)	0.013
	Female	552 (92.9%)	42 (7.1%)	594 (100%)	
Nationality	Saudi	1189 (95.0%)	63 (5.0%)	1252 (100%)	0.786
	Non-Saudi	877 (94.7%)	49 (5.3%)	926 (100%)	
Age		55.3 ± 12.4	59.4 ± 11.9	55.5 ± 12.4	<0.001
Weight		74.5 ± 15.5	71.6 ± 14.0	74.3 ± 15.4	0.052
Smoker		758 (95.8%)	33 (4.2%)	791 (100%)	0.122
Non-smoker		1308 (94.3%)	79 (5.7%)	1387 (100%)	
**Comorbidities**					
Dyslipidemia	Yes	218 (97.3%)	6 (2.7%)	224 (100%)	0.078
	No	1848 (94.6%)	106 (5.4%)	1954 (100%)	
Obesity	Yes	463 (95.3%)	23 (4.7%)	486 (100%)	0.643
	No	1603 (94.7%)	89 (5.3%)	1692 (100%)	
Diabetes mellitus	Yes	1266 (93.9%)	82 (6.1%)	1348 (100%)	0.011
	No	800 (96.4%)	30 (3.6%)	830 (100%)	
Hypertension	Yes	1376 (94.4%)	82 (5.6%)	1458 (100%)	0.147
	No	690 (95.8%)	30 (4.2%)	720 (100%)	
Cerebrovascular accident	Yes	104 (92.9%)	8 (7.1%)	112 (100%)	0.325
	No	1962 (95.0%)	104 (5.0%)	2066 (100%)	
Peripheral vascular disease	Yes	36 (92.3%)	3 (7.7%)	39 (100%)	0.467
	No	2030 (94.9%)	109 (5.1%)	2139 (100%)	
Carotid disease	Yes	29 (87.9%)	4 (12.1%)	33 (100%)	0.067
	No	2037 (95.0%)	108 (5.0%)	2145 (100%)	
Chronic obstructive pulmonary disease	Yes	86 (95.6%)	4 (4.4%)	90 (100%)	0.759
	No	1980 (94.8%)	108 (5.2%)	2088 (100%)	
**New York Heart Association (NYHA) Classification**					
0		12 (100%)	0 (0.0%)	12 (100%)	<0.001
I		11 (78.6%)	3 (21.4%)	14 (100%)	
II		1364 (96.3%)	52 (3.7%)	1416 (100%)	
III		576 (93.4%)	41 (6.6%)	617 (100%)	
IV		95 (85.6%)	16 (14.4%)	111 (100%)	
**Nature of the operation**					
Elective		1825 (96.4%)	68 (3.6%)	1893 (100%)	<0.001
Urgent		7 (70.0%)	3 (30.0%)	10 (100%)	
Emergency		232 (85.0%)	41 (15.0%)	273 (100%)	
**Other parameters**					
Creatinine clearance		81.6 ± 33.4	58.0 ± 30.4	80.3 ± 33.6	<0.001
Euroscore II		2.5 (2.1–4.0)	6.2 (2.9–12.0)	2.6 (2.1–4.1)	<0.001
Ejection fraction		45.2 ± 10.3	41.5 ± 12.1	45.1 ± 10.4	<0.001

**Table 4:** Cox regression proportional hazard analysis to predict risk factors associated with time to all-cause mortality (full cohort—multi-variate-adjusted hazard ratios).

Factors	Beta Coefficient	*p*-Value	Hazard Ratio (HR)	95.0% CI for HR	
				Lower	Upper
Euroscore II	0.03	0.015	1.03	1.01	1.05
Prolonged ventilation	1.44	0.000	4.23	2.18	8.24
Reintubation	1.52	0.000	4.56	2.58	8.06
Acute kidney injury	1.22	0.000	3.39	1.85	6.20
Continuous renal replacement therapy	0.78	0.006	2.19	1.25	3.84
Mediastinitis	1.95	0.059	7.06	0.93	53.54
Postoperative intra-aortic balloon pump (IABP)	1.08	0.000	2.94	1.73	5.00
Postoperative ECMO	0.90	0.012	2.46	1.22	4.94
Total Postoperative stay	−0.09	0.000	0.92	0.90	0.93

**Table 5:** Logistic regression to estimate association between Saudi and non-Saudi patients with covariate adjustments and use of propensity score matching techniques.

Factors	B	*p*-Value	Odds Ratio	95% C.I. for Odds Ratio	
				Lower	Upper
Nationality (Non-Saudi)	0.791	0.048	2.205	1.006	4.834
Propensity Scores	0.654	0.486	1.922	0.306	12.085

## Data Availability

The data will be made available with the corresponding authors when requested.
